# Protein Kinase CK2 Controls Ca_V_2.1-Dependent Calcium Currents and Insulin Release in Pancreatic β-cells

**DOI:** 10.3390/ijms21134668

**Published:** 2020-06-30

**Authors:** Rebecca Scheuer, Stephan Ernst Philipp, Alexander Becker, Lisa Nalbach, Emmanuel Ampofo, Mathias Montenarh, Claudia Götz

**Affiliations:** 1 Department of Medical Biochemistry and Molecular Biology, Saarland University, Kirrberger Str., bldg. 44, D-66424 Homburg, Germany; rebecca.gross@hotmail.de (R.S.); mathias.montenarh@uks.eu (M.M.); 2 Department of Experimental and Clinical Pharmacology and Toxicology, Saarland University Kirrberger Str., bldg. 45-46, D-66424 Homburg, Germany; stephan.philipp@uks.eu (S.E.P.); AlexBecker5683@hotmail.de (A.B.); 3 Institute for Clinical & Experimental Surgery, Saarland University Kirrberger Str., bldg. 65, D-66424 Homburg, Germany; lisa.nalbach@uks.eu (L.N.); emmanuel.ampofo@uks.eu (E.A.)

**Keywords:** CK2, INS-1, CX-4945, Ca_V_2.1, insulin secretion

## Abstract

The regulation of insulin biosynthesis and secretion in pancreatic β-cells is essential for glucose homeostasis in humans. Previous findings point to the highly conserved, ubiquitously expressed serine/threonine kinase CK2 as having a negative regulatory impact on this regulation. In the cell culture model of rat pancreatic β-cells INS-1, insulin secretion is enhanced after CK2 inhibition. This enhancement is preceded by a rise in the cytosolic Ca^2+^ concentration. Here, we identified the serine residues S_2362_ and S_2364_ of the voltage-dependent calcium channel Ca_V_2.1 as targets of CK2 phosphorylation. Furthermore, co-immunoprecipitation experiments revealed that Ca_V_2.1 binds to CK2 in vitro and in vivo. Ca_V_2.1 knockdown experiments showed that the increase in the intracellular Ca^2+^ concentration, followed by an enhanced insulin secretion upon CK2 inhibition, is due to a Ca^2+^ influx through Ca_V_2.1 channels. In summary, our results point to a modulating role of CK2 in the Ca_V_2.1-mediated exocytosis of insulin.

## 1. Introduction

The production and secretion of insulin from the β-cells of pancreatic islets is crucial for the regulation of glucose homeostasis. In response to changes in the level of blood glucose, the production and secretion of insulin is tightly regulated. This regulation is achieved by the opening and closing of ion channels, by intracellular signalling, by transcription and by post-transcriptional events [1]. There is increasing evidence that the protein kinase CK2 is strongly implicated in this regulation (for review see [2]). CK2, formerly known as casein kinase 2, is a highly conserved and ubiquitously expressed serine/threonine protein kinase, which is localized in nearly every compartment of the cell [3]. It is active as a tetrameric holoenzyme composed of two catalytic α- or α’- subunits and two non-catalytic β-subunits and also as α- or α’- subunits alone [4,5]. CK2 is not responsible for the regulation of one particular pathway but it seems to fine-tune various signaling pathways including the NFκB pathway, STAT3-, PTEN/PI3K/Akt- and the Wnt/β-catenin pathways [6,7,8,9,10,11]. Therefore, it is not surprising that CK2 is implicated in the regulation of stress response, unfolded protein response [12,13], DNA damage response [14], angiogenesis [15] and in cell development and differentiation [16]. Furthermore, CK2 is also implicated in the regulation of the carbohydrate metabolism [17]. Over a couple of years, there is increasing evidence that CK2 phosphorylates ion channels in the plasma membrane, which regulates the opening or closing of these channels [18,19,20,21]. Among these CK2 phosphorylated channels is the L-type channel Ca_V_1.2 [22,23]. In the present study, we continued our studies on CK2 in the regulation of insulin production in pancreatic β-cells by analyzing the role of CK2 in the phosphorylation and regulation of the Ca_V_2.1 channel. This channel belongs to the class of P/Q-type channels. Whereas L- and T-type channels were suggested to participate in the generation of action potentials, the P/Q-type channel is critically involved in the exocytosis of insulin from storage granules, especially at low glucose concentrations (6 mmol/l) [24]. It was shown that P/Q-type Ca^2+^ channels account for 45% of the whole-cell Ca^2+^ current during glucose-stimulated insulin secretion and that a blockade of P/Q-type Ca^2+^ channels suppressed exocytosis by ~80% [24].

Here, we describe the pore-forming α-subunit of the P/Q-type channel Ca_V_2.1 as a substrate for protein kinase CK2. Upon inhibition of CK2 kinase activity by the specific inhibitor CX-4945, insulin secretion and the glucose-induced rise in the cytosolic Ca^2+^ concentration is enhanced in pancreatic β-cells. In cells with strongly reduced expression of Ca_V_2.1, the CX-4945-induced rise in cytosolic Ca^2+^, as well as the enhanced insulin secretion, were considerably repressed. Thus, we conclude that the phosphorylation of Ca_V_2.1 channels by CK2 at least partially inhibits Ca^2+^ influx and the subsequent exocytosis of insulin.

## 2. Results

Recently, we [25,26] and others [27] described that, in pancreatic β-cells (MIN6, βTC3, isolated murine islets), insulin secretion is enhanced upon the inhibition of CK2 activity. Insulin secretion is triggered by cytosolic Ca^2+^, which is essential for the exocytosis of insulin storage granules. Therefore, here, we asked whether CK2 may be involved in the regulation of Ca^2+^ currents in β-cells of the pancreas. We decided to start with the analysis of the P/Q-type channel Ca_V_2.1, since this channel is critically involved in the exocytosis of insulin from granules from human pancreatic β-cells [24]. In an initial experiment, we asked whether this channel might be phosphorylated by CK2.

### 2.1. CK2 Phosphorylates Ca_V_2.1 at Serine Residues S_2362_ and S_2364_

**Figure 1:**
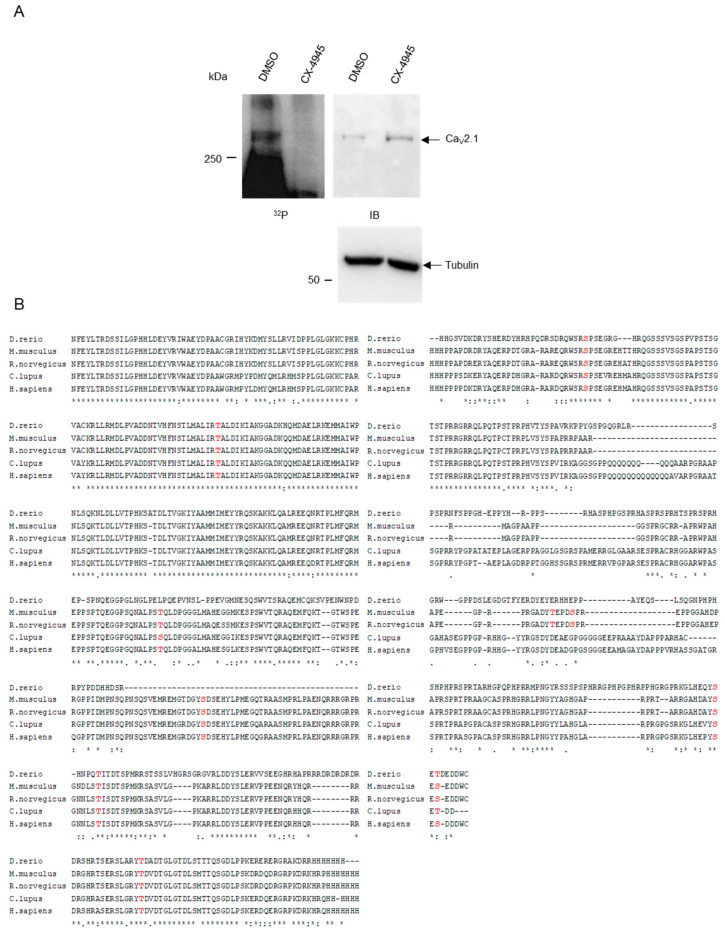

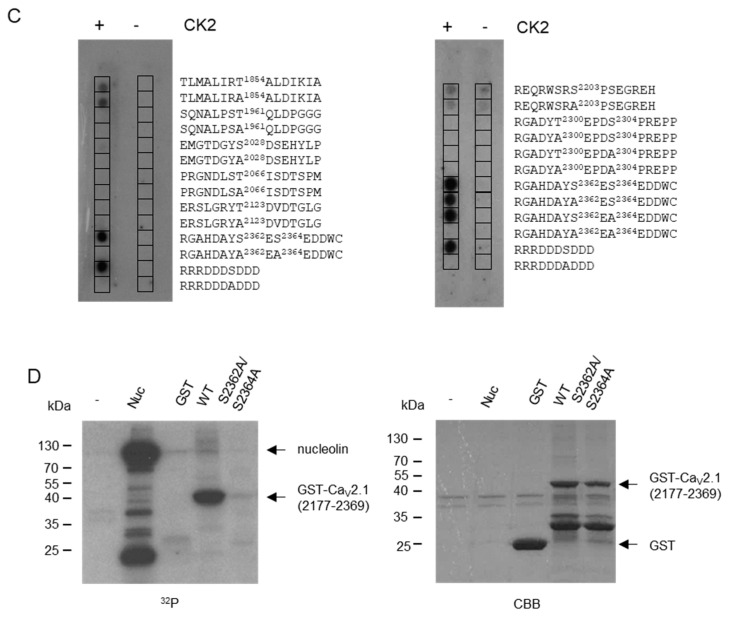
CK2 phosphorylates Ca_V_2.1. (**A**) An in vitro phosphorylation assay was carried out by incubating lysates (80 µg) of INS-1 cells with CK2 in the presence of DMSO (control) or 10 µM CX-4945. Proteins were subjected to SDS polyacrylamide gel electrophoresis and blotted onto a PVDF membrane. A representative autoradiography (^32^P) of the blotted proteins and the corresponding immunoblot (IB) of the same membrane with an anti-Ca_V_2.1 and an anti-tubulin-specific antibody is shown. (**B**) Comparison of the amino acid sequences of the Ca_V_2.1 C-terminus from different species (GenBank protein IDs: zebra fish NP_001315637.1, mouse AAW56205.1, rat XP_017456671.1, dog XP_013977279.1, human AAB64179.1). Dashes indicate a missing amino acid and spacing has been adjusted to maximize the homology of the sequences. The highlighted amino acids indicate putative CK2 phosphorylation sites with the sequence S/TxxD/E (**C**) Peptides with the putative CK2 phosphorylation sites shown in B (based on the murine sequence) were spotted on a cellulose membrane. Filters were incubated in the absence (-) or the presence of CK2 (+) and [^32^P]γATP. A representative autoradiography is shown. (**D**) An in vitro phosphorylation assay was carried out by incubating equal amounts of GST, GST–Ca_V_2.1 (2177-2369) wild-type or double mutant S2362A/S2364A with CK2 and [^32^P]γATP. A sample with CK2 alone (-) or with nucleolin, as an established CK2 substrate, served as controls. Samples were analyzed on a 12.5% SDS polyacrylamide gel and subjected to staining with Coomassie Brilliant Blue (CBB) followed by autoradiography (^32^P). A representative autoradiography and the corresponding CBB staining is shown.

As a source for an initial phosphorylation experiment with the full-length Ca_V_2.1 protein, we used equal aliquots of a total protein lysate of rat pancreatic β-cells (INS-1 832/13, subsequently called INS-1). We added CK2 and [^32^P]γATP either in the presence of the specific CK2 inhibitor CX-4945 or with DMSO as a solvent control. After phosphorylation, proteins were separated by SDS polyacrylamide gel electrophoresis, blotted to a PVDF membrane and subjected to autoradiography (Figure 1A, left panel, ^32^P). Subsequently, the same membrane was subjected to an immunoblot analysis with a Ca_V_2.1 antibody and, as loading control, with an anti-tubulin antibody (Figure 1A, right panel, IB). In the autoradiography, we detected a protein with the expected molecular weight of about 270 kDa, which migrated at the same position as the protein detected by immunoblot analysis. Under the influence of the CK2 inhibitor, the phosphorylation signal disappeared in the autoradiography, although the protein was still present, as shown in the immunoblot. The detection of tubulin in both lanes showed the equal loading of the extracts. Thus, we demonstrated that the endogenous Ca_V_2.1 protein from INS-1 cells is a substrate of protein kinase CK2.

Since CK2 is a mostly intracellular-acting kinase, and a yeast two-hybrid screen revealed an interaction between the C-terminus of Ca_V_2.1 and the regulatory CK2β subunit [28], we compared the C-termini of the Ca_V_2.1 channel α_1A_ subunits (CACNA 1a) of zebra fish (Genbank protein ID: NP_001315637.1), mice (AAW56205.1), rats (XP_017456671.1), dogs (XP_013977279.1) and humans (AAB64179.1, 87% identity) and searched for consensus sequences of protein kinase CK2 phosphorylation sites (Figure 1B). Within the cytosolic C-terminus of the murine Ca_V_2.1 protein from amino acid residues 1764–2369, we found 10 putative CK2 phosphorylation sites corresponding to the minimal consensus sequence S/TxxD/E [29,30] (Figure 1B). Six of them were conserved between the fish, mouse, rat, dog and human proteins.

To identify CK2 phosphorylation site(s) within the Ca_V_2.1 protein, we performed a dotblot analysis (Figure 1C). Pentadecapeptides corresponding to the putative CK2 phosphorylation sites with the serine/threonine residues to be phosphorylated in the centre of the peptide were synthesized on a cellulose membrane. We compared wild-type peptides to peptides with an exchange of serine/threonine to a non-phosphorylatable alanine residue. Two membranes with the spotted CK2 peptides and a commonly used control peptide (RRRDDDSDDD) were each incubated either with protein kinase CK2 and radiolabeled [^32^P]γATP (+) or with [^32^P]γATP alone as a negative control (-). The control peptides showed clear signals that were absent when the serine or threonine residue was replaced by alanine (Figure 1C). We also detected faint signals for the peptides containing T_1854_ and S_2203_. However, these signals were observed in both the wild-type and the mutant peptide or even in the absence of the CK2 holoenzyme and might have originated from the phosphorylation of a non-canonical phosphorylation site or from the unspecific binding of ATP. Thus, we assessed these signals to be not relevant. In contrast, we observed an unambiguous phosphorylation of the residues S_2362_ and S_2364_. Both sites seemed to be phosphorylated equally well by CK2, since only the double mutant, but no single mutant, lacked the phosphorylation.

To verify these results in an independent approach, we subcloned the C-terminal fragments of murine Ca_V_2.1, encompassing the amino acid residues 2177–2369, either as its wild-type or as its double-mutated (S_2362_A/S_2364_A) version, in frame with glutathione-S-transferase (GST) tags. The recombinant fusion proteins were expressed in *Escherichia coli*, purified by affinity chromatography, subjected to in vitro phosphorylation with the CK2 holoenzyme, separated by SDS gel electrophoresis and analyzed by autoradiography (Figure 1D). The phosphorylation of the well-established CK2 substrate nucleolin served as a positive control. The unfused GST protein was used to exclude that the GST sequence was phosphorylated (negative control). We observed a distinct signal for the GST–Ca_V_2.1_(2177-2369)_ fragment at the predicted size of 46 kDa. The fragment was only efficiently phosphorylated in its wild-type but not its double-mutated form, thus confirming the data from the peptide filter and demonstrating that S_2362_ and S_2364_ are target sites of CK2 phosphorylation within the C-terminus of Ca_V_2.1.

### 2.2. CK2 Interacts with C-Terminal Residues of Ca_V_2.1.

**Figure 2:**
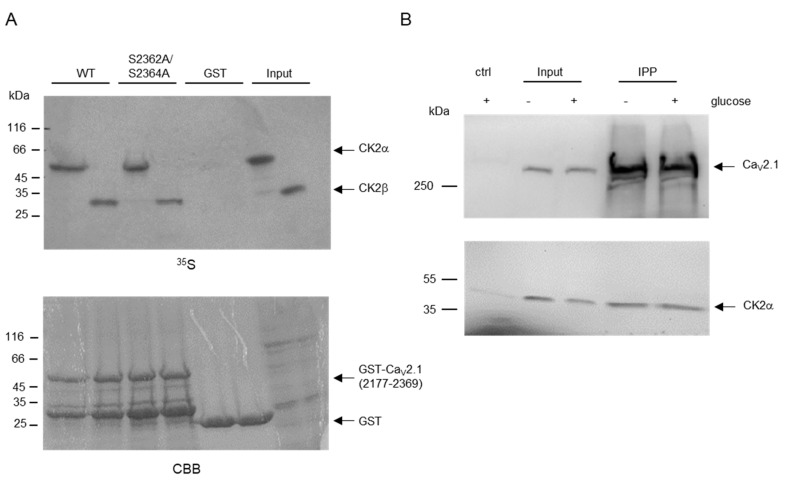
CK2 binds to Ca_V_2.1. (**A**) GST pull-down analysis of Ca_V_2.1 and CK2. Equal amounts of GST–Ca_V_2.1 (2177-2369) wild-type and double-mutant S_2362_A/S_2364_A coupled to GSH sepharose were incubated with in vitro-translated and [^35^S]-methionine-labeled CK2α and CK2β proteins. Proteins bound to the affinity matrix were subjected to SDS polyacrylamide gel electrophoresis, stained with Coomassie Brilliant Blue (CBB), followed by autoradiography (^35^S). A representative autoradiography and a corresponding CBB staining are shown. (**B**) Co-immunoprecipitation of Ca_V_2.1 and CK2 from INS-1 cells. Five milligrams of extract from cells grown with 0 mM (-) or 10 mM (+) glucose were pre-cleared twice from non-specific binding proteins with protein G sepharose beads (ctrl). After the centrifugation of the beads, the supernatants were incubated with a rabbit Ca_V_2.1 antibody coupled to protein G sepharose overnight. Bound proteins were extracted with sample buffer, separated in a 10% SDS polyacrylamide gel and blotted to a PVDF membrane. Ca_V_2.1 and CK2α subunit were detected with a rabbit monoclonal antibody directed against Ca_V_2.1 or the mouse monoclonal antibody 1A5 against CK2α. Ctrl: proteins bound to protein G sepharose; input: 2% of whole protein extract used for immunoprecipitation; IPP: proteins immunoprecipitated with Ca_V_2.1-specific antibody.

CK2 interacts strongly with its substrates [31]. The catalytic α-subunit binds the substrates with its substrate binding domain between residues D175 and E201. For the right orientation of the substrate, however, some substrates are targeted by the regulatory β-subunit as well [32], as it has also been suggested for the C-terminus of Ca_V_2.1 after yeast two-hybrid experiments [28]. Therefore, we analyzed, in pull-down experiments, whether both subunits might interact with Ca_V_2.1. The individual CK2α and CK2β subunits were labeled with [^35^S]-methionine by in vitro translation and mixed separately with the GST-tagged Ca_V_2.1(2177-2369) fusion protein in its wild-type and its double-mutated (S_2362_A/S_2364_A) forms. Protein complexes were pulled down using a glutathione (GSH) sepharose affinity matrix and analyzed on a SDS polyacrylamide gel. GST fusion proteins were stained with Coomassie Brilliant Blue (CBB) and bound CK2 subunits were visualized by autoradiography (Figure 2A). Both subunits interacted with the Ca_V_2.1 C-terminal fragment. The exchange of the serine 2362 and 2364 residues to alanine had no impact on the binding capacity for CK2. Thus, we could demonstrate that both subunits of CK2 were able to bind Ca_V_2.1 and that binding is independent of CK2 phosphorylation.

Next, we checked whether we could also detect such an interaction in INS-1 cells and whether there might be an influence of glucose on such an interaction. Therefore, we starved the cells for 2 h in 0 mM glucose and cultivated the cells for another 3 h in either 0 mM or 10 mM glucose. Cells were lysed and Ca_V_2.1 protein was immunoprecipitated using anti-Cav2.1 antibody. Precipitated proteins were detected by immunoblot with either anti-Ca_V_2.1 or anti-CK2α antibodies (Figure 2B). Under both glucose conditions, Ca_V_2.1 was precipitated equally well and in each precipitate we detected comparable amounts of co-immunoprecipitated CK2α subunits. Thus, Ca_V_2.1 and CK2α interact in pancreatic INS-1 β-cells; however, the interaction was obviously not dependent on glucose.

### 2.3. Inhibition of CK2 Activity in INS-1 Cells Leads to a Rise in Cytosolic Ca^2+^-Concentration.

**Figure 3:**
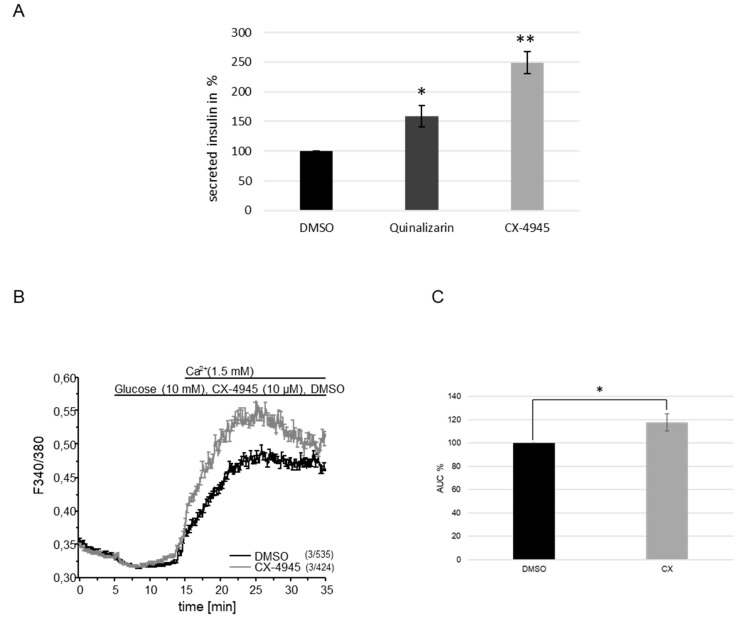
Inhibition of CK2 increases insulin secretion and cytosolic Ca^2+^ concentration in INS-1 cells. Prior to the experiments, cells were starved for 2 h in glucose-free medium. (**A**) Glucose-induced insulin secretion in the absence or presence of the CK2 inhibitors CX-4945 (10 µM) or quinalizarin (50 µM). Insulin levels in the supernatant were measured after 30 min. Data are expressed as means of three independent experiments with two technical replicates each. Statistical analysis was performed by using Student’s *t*-tests. * *p* < 0.05, ** *p* < 0.01. (**B**) Ca^2+^ imaging experiments in the absence or in the presence of the CK2 inhibitor CX-4945 (10 µM). Measurements were started in Ca^2+^-free medium. Changes in the cytosolic Ca^2+^ concentration were determined by Fura 2-AM (5 µM) imaging measurements and plotted versus time. Each trace represents the mean ± SEM of the fluorescence ratios (F_340/_F_380_*)* obtained in three independent experiments of the total number of cells indicated in brackets. (**C**) Mean area under the curve (AUC) of the three independent experiments shown in B. AUC of the DMSO-treated cells was set as 100%. Statistical analysis was performed by using Student’s *t*-tests. * *p* < 0.05.

Insulin secretion from storage granules is triggered by the rise in the cytosolic Ca^2+^ concentration. Formerly, we described that insulin secretion is enhanced upon the inhibition of CK2 activity with specific inhibitors in MIN6 cells [25]. Now, we analyzed whether this also applies to INS-1 cells. INS-1 cells were exposed to a glucose stimulus and simultaneously treated with the CK2 inhibitors CX-4945, quinalizarin or DMSO as a control for 30 min. We determined the amount of secreted insulin in the cell culture medium (Figure 3A). Over 30 min, the amount of insulin accumulated in the medium of control cells (DMSO) to 20 ng/mL insulin (corresponding to 100%). Using quinalizarin, we observed a roughly 1.5-fold increase in insulin in the medium. Cells which had been treated with CX-4945, however, secreted more than double the amount of control cells. Thus, in line with our previous results obtained with MIN6 cells, INS-1 cells showed increased insulin secretion in the presence of protein kinase CK2 inhibitors.

Having shown an increase in insulin secretion from pancreatic β-cells after the inhibition of CK2 activity, we asked whether CK2 also influences the intracellular Ca^2+^ concentration. For that purpose, INS-1 cells were cultured in a KRBH buffer free of glucose and Ca^2+^ for 30 min. During that time, the cells were loaded with the fluorescent Ca^2+^ indicator dye Fura 2-AM and recordings of the cytosolic Ca^2+^ concentration were started in glucose- and Ca^2+^-free media. After five minutes, cells were incubated with medium containing 10 mM glucose and 10 μM CX-4945 or DMSO as a control. Another 5 min later, 1.5 mM Ca^2+^ was added. As shown in Figure 3B, after Ca^2+^ re-addition, we found a strong increase in the cytosolic Ca^2+^ concentration that was strongly enhanced in the presence of CX-4945, indicating that the inhibition of CK2 increases the Ca^2+^ entry from outside into the cell.

### 2.4. The Rise in Cytosolic Ca^2+^ after Pharmacological Inhibition of CK2 is Dependent on the Presence of Ca_V_2.1.

**Figure 4:**
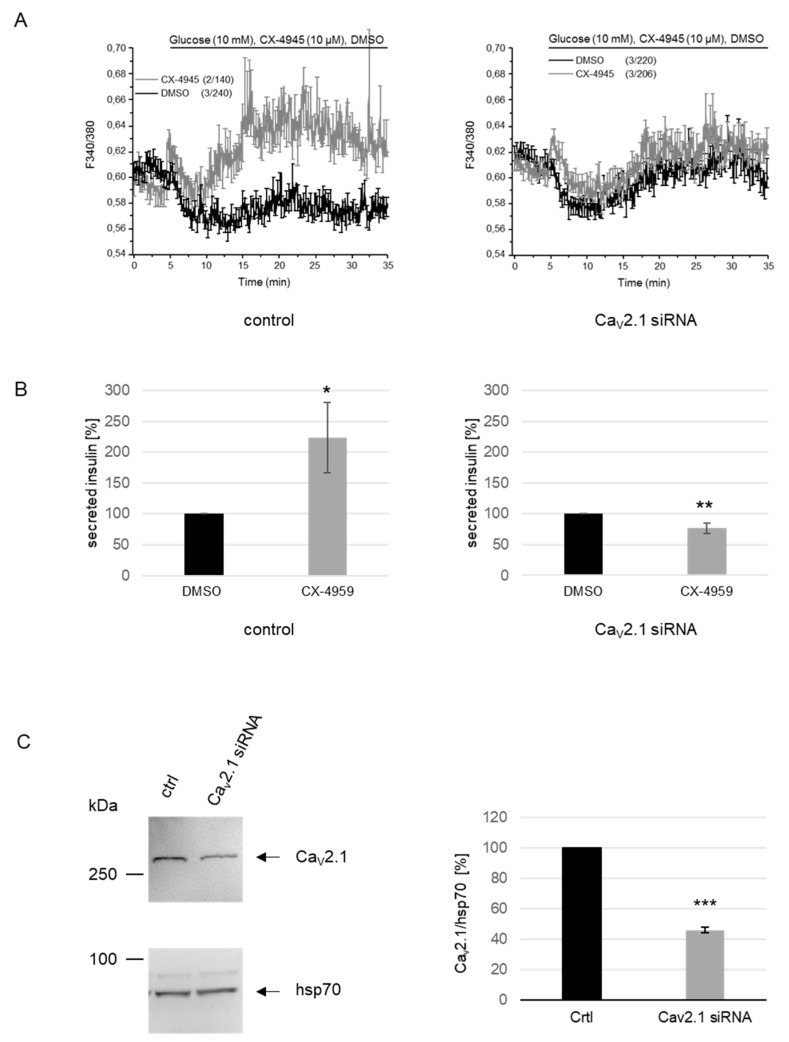
Knockdown of Ca_V_2.1 expression abolishes the CX-4945-induced increase in the cytosolic Ca^2+^ concentration and enhanced insulin secretion in INS-1 cells. (**A**–**C**): INS-1 cells were transfected with Ca_V_2.1 siRNA or scrambled control siRNA (200 nM), together with the fluorescent transfection indicator siGlo for 48 h. (**A**) Ca^2+^ imaging experiments after Ca_v_2.1 knockdown. Prior to the measurement, the cells were starved for 2 h in glucose-free medium. Cells were then incubated with the CK2 inhibitor CX-4945 (10 µM) or the solvent DMSO as a control and insulin secretion was induced with glucose (10 mM). Calcium imaging was only done in those cells where the successful transfection was indicated by siGlo. Changes in [Ca^2+^] were determined by Fura 2-AM (5 µM) measurements and plotted versus time. Each trace represents the mean ± SEM of fluorescence ratios (*F_340/380_)* of two or three independent experiments of the total number of cells indicated in brackets. (**B**) Cell culture supernatants of cells treated as described for A were collected and analyzed for secreted insulin by an ELISA assay. Determination was done in three independent experiments with two technical replicates each and values were normalized to the respective DMSO control. Data are expressed as means ± SEM. Statistical analysis was performed by using Student’s *t*-tests. * *p* < 0.05, ** *p* < 0.01. (**C**) Equal amounts of extracts from all cells were analyzed by SDS polyacrylamide gel electrophoresis and subsequent immunoblot analysis with anti-Ca_V_2.1- or anti-hsp70-specific antibodies (left panels). A representative immunoblot is shown. Ratios between the arbitrary amount of Ca_V_2.1 and the loading control hsp70 were quantified by densitometry (Ca_V_2.1/hsp70) and compared with the corresponding control value normalized to 100% (right panel). Data are expressed as means ± SEM of three independent experiments. Statistical analysis was performed by using Student’s *t*-tests. *** *p* < 0.001.

Since we have shown above that the Ca^2+^ channel Ca_V_2.1 is a substrate of CK2, we analyzed whether the CK2-dependent phosphorylation of the Ca_V_2.1 channel contributes to the modulation of Ca^2+^ entry. For that purpose, Ca_V_2.1 expression was silenced by RNA interference using Ca_V_2.1 siRNA. INS-1 cells were transfected with Ca_V_2.1 siRNA or scrambled siRNA as a control, together with siGlo as a transfection indicator and incubated in glucose-free medium for 2 h. After loading the cells with Fura 2-AM in glucose-free KRBH buffer containing 1.5 mM Ca^2+^, we started the calcium imaging experiments (Figure 4A). Five minutes later, we added 10 mM glucose, either in the presence or absence of CX-4945 and fluorescence signals were recorded for an additional 30 min.

Under these conditions in control cells, we observed only a slight increase in the cytosolic Ca^2+^ concentration after the addition of glucose, which again was strongly enhanced in the presence of CX-4945. However, in cells transfected with Ca_V_2.1 siRNA, there was essentially no CX-4945-dependent difference in the cytosolic Ca^2+^ concentration. To analyze the impact of the down-regulation of Cav2.1 on insulin secretion, we collected cell culture media of cells transfected with either scrambled siRNA or with Ca_V_2.1 siRNA and treated them simultaneously with DMSO or CX-4945 to determine the insulin content. Figure 4B shows the normalized values of secreted insulin. In the control experiment with scrambled siRNA, insulin secretion was enhanced by more than twofold, as already shown in Figure 3A; in contrast, cells which were transfected with Ca_V_2.1 siRNA secreted a comparable level of insulin, independent of the treatment with CX-4945. After calcium imaging, we harvested the cells and analyzed the success of knocking down Ca_V_2.1. Equal amounts of extracts of all cells treated with scrambled or Ca_V_2.1 siRNA were subjected to an immunoblot analysis with a Ca_V_2.1 and an hsp70 antibody (Figure 4C). There was a considerable reduction in the level of Ca_V_2.1 protein compared to cells transfected with control siRNA. Thus, we conclude that the CK2-dependent phosphorylation of the Ca_V_2.1 channel indeed contributes to the modulation of Ca^2+^ entry and to the subsequent insulin secretion.

## 3. Discussion

Previous experiments have shown that protein kinase CK2 is implicated in the regulation of insulin production and secretion in pancreatic β-cells. An active CK2 is likely to act as a negative regulator of the function of a pancreatic β-cell, since its inhibition improves insulin transcription and secretion [25,26,27]. We have already identified several substrates which have an impact on the transcription machinery of the β-cell. The key transcription factor pancreatic and duodenal homeobox-1 (PDX-1) and the upstream transcription factors USF1 and USF2 are among various transcription factors which are implicated in the regulation of insulin production in pancreatic β-cells [33,34]. We have previously shown that PDX-1 and USF1 are substrates of protein kinase CK2 and that the phosphorylation of these two transcription factors by CK2 is adversely implicated in the regulation of insulin production and secretion [25,26,35,36,37,38]. In light of these results, it is remarkable that CK2 expression is enhanced in a murine diabetic model and even in the sera of type 2 diabetes mellitus patients [39]. Iori et al. [40] observed a higher dependence of fibroblasts from type 1 diabetes mellitus patients on CK2 although there was no change in expression and activity compared to normal cells.

A prerequisite of glucose-stimulated insulin secretion is the uptake of extracellular Ca^2+^ by voltage-gated calcium channels, thus leading to a rise in the intracellular calcium concentration. L-type calcium channels play a predominant role in the insulin secreting β-cells ([41] and literature cited therein). However, studies using the P/Q-type channel blocker ω-agatoxin IV A showed that, in rat β-cells, high voltage Ca^2+^ currents during glucose-stimulated insulin secretion were blocked with an average reduction of 48.4 ± 8.5% and the subsequent insulin secretion was reduced by 30% [42]. In human β-cells, about 25% of the currents were contributed by the P/Q-type channel [43]. The only P/Q-type calcium channel, consisting of the pore-forming subunit α_1A_ and variable auxiliary β, γ and α_2_δ subunits, is Ca_V_2.1 [44]. Cav2.1 was shown to be associated with type 2 diabetes [45].

We identified the pore-forming α_1A_ subunit as a substrate of protein kinase CK2 and mapped the phosphorylated sites to two residues of the cytosolic C-terminus of Ca_V_2.1 at S_2362_ and S_2364_. It is known that, in addition to the minimal consensus sequence S/TxxD/E, CK2 prefers sites with flanking acidic residues or at least acidic amino acids between the phosphorylation target and the acidic residue at position +3 [46] and that applies to the two identified amino acid residues. With one exception (S_2028_), the other given putative sites are not embedded in an acidic environment. In the present paper, we demonstrated that endogenous Ca_V_2.1 from INS-1 cells is phosphorylated by CK2; however, we could not prove that the phosphorylated sites were identical with those from the in vitro experiments. However, both sites were found to be phosphorylated in the course of a phosphoproteome analysis in 3T3-L1 cells [47]. This observation, together with the fact that the CK2β subunit was found as a binding partner of the C-terminal region of Ca_V_2.1 in a yeast two-hybrid screen of a human brain cDNA library [28], strengthens the assumption that this phosphorylation is of physiological significance for the regulation of Ca_V_2.1 channels.

Protein subunits, such as the 165 kDa and the 55 kDa subunits of the L-type Ca^2+^ channels Ca_V_1.1 and Ca_V_1.2, are also phosphorylated by CK2 [48]. Threonine 1704 is presumably phosphorylated by CK2 and it was suggested that this phosphorylation has an important role for the basal activity of Ca_V_1.2 [49]. During the differentiation of cardiomyocytes, Ca_V_1.2 is activated by angiotensin II mediated signaling. The full activation of Ca_V_1.2 is reached by phosphorylation at threonine 1704 in a CK2α’-dependent manner, which leads to the dissociation of an autoinhibitory complex of the Ca_V_1.2 cytosolic domain [23]. Mice with mutations at both sites, S_1700_ and T_1704_, have a greatly reduced basal L-type calcium current and a reduced response to β-adrenergic stimulation [49,50]. These mutant mice have an impaired contractile function, decreased exercise capacity and cardiac hypertrophy.

The cytoplasmic regions of the pore-forming Ca_V_2.1 subunit are responsible for channel regulation by interacting with regulatory proteins [51]. Among the regulatory proteins, which bind to the C-terminal domain of Ca_V_2.1, is calmodulin (CaM) [51]. CaM acts as Ca^2+^ sensor and binds to the CaM binding domain (CBD) in the C-terminus of rat Ca_V_2.1 between amino acids 1969–2000 [52] and to a second motif between amino acids 1909–1949, which is analogous to the so-called IQ motif of Ca_V_1 channels [53]. The binding of CaM to both motifs contributes to the regulation of Ca_V_2.1 channel activity [54]. For a long time, it has been known that CaM is phosphorylated by CK2 [55,56]. CaM is a peculiar substrate because it is phosphorylated by CK2α but not by the CK2 holoenzyme consisting of CK2α and CK2β [57]. It remains to be elucidated how the inhibition of CK2 might regulate the interaction of CaM with Ca_V_2.1. The binding regions for CaM are far away from the CK2 phosphorylation sites at residues S_2362_ and S_2364_ of Ca_V_2.1, and therefore it seems to be rather unlikely that these phosphorylation sites participate in the regulation of the Ca_V_2.1/CaM interaction.

The extreme C-terminal part of Ca_V_2.1 represents an interacting platform with proteins of the so-called active zone (AZ) [58]. These interactions were proposed to control the coupling and abundance of Ca_V_2.1 in the presynaptic region; however, these results are controversial [59]. The PDZ domain of the Rab-interacting molecules 1/2 (RIM 1/2) and MINT1 interact with the DxWC motif, which is unique for its P/Q-type Ca_V_2.1 and N-type channels, but not L- and T-type channels [60,61]. The interaction between these proteins is presumably necessary for the localization of the channels to the active zone. The CK2 phosphorylation site directly precedes this motif and it is tempting to speculate that the phosphorylation may modulate the RIM 1/2 or MINT1 interaction.

In the present work, we analyzed the CK2-dependent change of the cytosolic Ca^2+^ concentration and the subsequent basal release of insulin using glucose as secretagogue. Both cytosolic Ca^2+^ and insulin release increased after the inhibition of CK2 activity. Using RNA interference, this basal increase in calcium content was clearly dependent on the presence of Ca_V_2.1. Whether the phosphorylation at serine residues S_2362_ and S_2364_ by CK2 takes part in this regulation, potentially by modulating the interaction with docking proteins of the active zone, awaits further experiments.

## 4. Materials and Methods

### 4.1. Cell Culture and Treatment of Cells

The rat insulinoma cell line INS-1 832/13 [62] was cultured in RPMI 1640 medium containing 11 mM glucose (Life Technologies, Darmstadt, Germany) supplemented with fetal calf serum (FCS, 10%, Biochrom AG, Berlin, Germany), sodium pyruvate (1 mM) and β-mercaptoethanol (50 µM) at 37 °C in a humidified atmosphere containing 95% air and 5% CO_2_. Cells were seeded in six-well dishes the day before experiments. A 10 mM stock solution of the CK2 inhibitors CX-4945 (SelleckChem, Munich, Germany) or quinalizarin (Labotest OHG, Niederschöna, Germany), each dissolved in dimethyl sulfoxide (DMSO, Merck, Darmstadt, Germany), were diluted in experiments to a final concentration of 10 or 50 µM, respectively. Control experiments were performed with DMSO.

### 4.2. siRNA Transfection

Ca_V_2.1 siRNA (L-090177-02-0005) and negative control siRNA (D-001810-10-05) were obtained from Dharmacon (Lafayette, Colorado, USA). The transfection of siRNA was performed according to the supplier’s protocol with 200 nM siRNA using the transfection reagent DharmaFECT 1 (10 µL). Simultaneously, a transfection indicator (siGLO Green Transfection Indicator, Dharmacon, Lafayette, Colorado, USA, D-001630-01-05) was transfected according to the manual. The absorbance/emission maximum of siGLO is 494/520 nm, an FITC filter was used for the detection. Calcium imaging experiments were only done with cells which were identified by this fluorescent transfection indicator. The INS-1 832/13 cells were then incubated in a 37 °C humidified incubator with 5% CO_2_ for 48 h.

### 4.3. Immunoblot

The extraction of proteins, SDS polyacrylamide gel electrophoresis and western blot analysis were essentially done as described by Servas et al. [63]. For the detection of the Ca_V_2.1 protein, we used a specific antibody from Abcam (ab181371, 1:1000, monoclonal rabbit antibody). Protein kinase CK2 was detected with antibody 1A5 (α-subunit, 1:250, monoclonal mouse antibody) [64]. Furthermore, we used an hsp70-specific antibody (Acris Antibodies GmbH, Herford, Germany) or an α-tubulin antibody (clone DM1A, Sigma-Aldrich, Munich, Germany) as a loading control.

### 4.4. Immunoprecipitation

After the starvation of INS-1 832/13 for 2 h in glucose-free RPMI 1640 medium, cells were incubated with glucose at different concentrations (0 mM and 10 mM) for 3 h. Cells were extracted and 5 mg of total protein were subjected to immunoprecipitation. The cell lysates were pre-cleared twice, using protein G sepharose (GE Healthcare, Freiburg, Germany) for 1 h at 4 °C. Ca_V_2.1 antibodies (Abcam, Cambridge, UK, ab181371, 10 µL), pre-incubated with protein G sepharose, were used for the immunoprecipitation of proteins from the pre-cleared lysates. After an overnight incubation at 4 °C, the supernatant was removed, beads were washed three times with PBS and bound proteins were eluted with SDS sample buffer. Proteins were separated on a 7.5% SDS polyacrylamide gel and detected by immunoblot with antibodies for Ca_V_2.1 (ab181371) or CK2α (1A5).

### 4.5. Insulin Secretion

The determination of secreted insulin was essentially done as described by Merglen et al. [65]. Briefly, INS-1 832/13 cells were maintained for 2 h in glucose-free RPMI 1640 medium (Life Technologies, Darmstadt, Germany). Cells were incubated in glucose-free Krebs–Ringer bicarbonate HEPES buffer (KRBH: 135 mM NaCl, 3.6 mM KCl, 5 mM NaHCO_3_, 0.5 mM NaH_2_PO_4_, 0.5 mM MgCl_2_, 1.5 mM CaCl_2_ and 10 mM HEPES, pH 7.4) supplemented with 0.1% BSA for 30 min. After the incubation, cells were washed with glucose-free KRBH and then incubated with KRBH containing 10 mM glucose and the CK2 inhibitor CX-4945 (10 µM, SelleckChem, Munich, Germany) or quinalizarin (50 µM, Labotest OHG, Niederschöna, Germany) for 30 min. Supernatants were collected and insulin secretion was measured by ELISA according to the manufacturer’s instructions (Rat Insulin ELISA Kit, Fisher Scientific, Waltham, Massachusetts, USA).

### 4.6. Calcium Imaging

INS-1 832/13 cells coated on glass coverslips were maintained for 2 h in glucose-free RPMI 1640 medium (Life Technologies, Darmstadt, Germany) and then loaded with 5 μM Fura-2-acetoxymethyl ester (Fura-2AM, Fisher Scientific, Waltham, Massachusetts, USA) dissolved in glucose-free KRBH supplemented with 0.1% BSA for 30 min at 37 °C. After loading, cells were washed with glucose-free KRBH and analyzed in 300 µL glucose-free KRBH in an open chamber using a Polychrome V-based imaging system (TILL Photonics, Martinsried, Germany) equipped with an inverted microscope (Axiovert S100, Zeiss, Oberkochen, Germany). Fura-2 was excited every 3 s at wavelengths (λ) of 340 nm and 380 nm for 20 ms and fluorescence emissions of λ > 510 nm were captured using a 20× Fluar objective (Zeiss, Oberkochen, Germany) and a TILL IMAGO CCD camera (TILL Photonics). After imaging for approximately 5 min to establish baseline conditions, 300 µL KRBH containing 20 mM glucose and 20 µM CX-4945 (SelleckChem, Munich, Germany) were added manually to the solution. The ratio F340/F380 was calculated from F340 and F380 pictures after background correction, i.e., the subtraction of the fluorescence intensity at 340 and 380 nm excitation from a cell-free area. Single cells were marked as regions of interest and F340/F380 was plotted versus time. The monochromator, camera, acquisition and analysis were controlled by TILLvisION software (TILL Photonics). The Ca^2+^ re-addition experiment was started in Ca^2+^-free KRBH with Ca^2+^ being added after 15 min (Figure 3).

### 4.7. Plasmids

Ca_V_2.1 (pcDNA6) was a gift from Diane Lipscombe (Addgene plasmid # 26578) [66] and used for amplifying the Ca_V_2.1 sequence from codon 2177 to 2369 by PCR. The amplified sequences were cloned into the BamHI/EcoRI site (Ca_V_2.1 (2177-2369)) of the bacterial expression vector pGEX-4T-1 (GE Healthcare), generating a fusion construct with an N-terminal GST tag. Ca_V_2.1 phosphorylation mutants were created using a QuikChange site-directed mutagenesis kit (Stratagene, La Jolla, CA, USA) according to the manufacturer’s protocol. All constructs were verified by sequencing.

### 4.8. GST Pull-Down Assay

GST pull-down assays were essentially done as described in [63].

### 4.9. In Vitro Phosphorylation

For the analysis of the phosphorylation of Ca_V_2.1, peptides (15 amino acids) with putative CK2 phosphorylation sites were synthesized on a cellulose membrane (M. Jung, Homburg, Germany). The peptide filter was activated in methanol and incubated in kinase buffer (50 mM Tris-HCl, pH 7.5, 150 mM NaCl, 5 mM MgCl_2_, 1 mM DTT) supplemented with 1% BSA overnight at 4 °C. After incubation, the filter was washed with kinase buffer and the phosphorylation reaction was started by the addition of CK2 holoenzyme and [^32^P]γATP (10 µCi, Hartmann Analytic, Braunschweig, Germany) in 1 mL kinase buffer. As a negative control, the mixture without CK2 was tested on a second identical filter. The peptide filters were incubated for 1 h at room temperature with gentle shaking. After incubation, the filter was washed with kinase buffer, air dried and analyzed by autoradiography.

Recombinant GST-tagged Ca_V_2.1 proteins were mixed with a recombinant CK2 holoenzyme in 20 µL kinase buffer. The phosphorylation reaction was started by the addition of [^32^P]γATP (Hartmann Analytic, Braunschweig, Germany). After 30 min at 37 °C, the reaction was stopped by the addition of 10 µL sample buffer (130 mM Tris-HCl pH 6.8, 0.02% bromophenol blue, 10% β-mercaptoethanol, 20% glycerol, 4% (*w*/*v*) SDS). Proteins were separated on SDS polyacrylamide gel (10% or 12.5%) followed by Coomassie Brilliant Blue staining and autoradiography.

### 4.10. Statistical Analysis

Microsoft Excel 2013 and the software “Quantity One 1-D Analysis” (version 4.6.7) from Bio-Rad Laboratories Inc. (Feldkirchen, Germany) were used to analyze the data. All values were expressed as mean ± SEM of at least three independent experiments. Statistical analysis of the data was performed using the Student’s *t*-test (two-tailed, unpaired), statistically significant differences were shown as follows: *** *p* < 0.001, ** *p* < 0.01 or *p < 0.05.

## Abbreviations

CaM	Calmodulin
DMSO	Dimethyl sulfoxide
SDS	Sodium dodecylsulfate
PVDF	Polyvinylidene difluoride
KRBH	Krebs–Ringer bicarbonate HEPES buffer
